# S100 Proteins in the Pathogenesis of Psoriasis and Atopic Dermatitis

**DOI:** 10.3390/diagnostics13203167

**Published:** 2023-10-10

**Authors:** Natsuko Saito-Sasaki, Yu Sawada

**Affiliations:** Department of Dermatology, University of Occupational and Environmental Health, Kitakyushu 807-8555, Japan; natsuko-saito@med.uoeh-u.ac.jp

**Keywords:** S100 proteins, atopic dermatitis, psoriasis

## Abstract

The skin, the outermost layer of the human body, is exposed to various external stimuli that cause inflammatory skin reactions. These external stimulants trigger external epithelial cell damage and the release of intracellular substances. Following cellular damage or death, intracellular molecules are released that enhance tissue inflammation. As an important substance released from damaged cells, the S100 protein is a low-molecular-weight acidic protein with two calcium-binding sites and EF-hand motif domains. S100 proteins are widely present in systemic organs and interact with other proteins. Recent studies revealed the involvement of S100 in cutaneous inflammatory disorders, psoriasis, and atopic dermatitis. This review provides detailed information on the interactions among various S100 proteins in inflammatory diseases.

## 1. Introduction

The skin is the human body’s outermost layer organ; thus, it is exposed to a variety of external stimuli associated with inflammatory reactions [1,2,3]. These external stimulants trigger external epithelial cell damage and release intracellular substances [4,5]. Following cellular damage or death, intracellular molecules such as damage-associated molecular patterns (DAMPs) and alarmins are released to enhance tissue inflammatory reactions [6]. These substances serve as “danger signals” that alert neighboring cells to anomalies in the human body. The importance of DAMPs as inducers of inflammatory tissue reactions has been elucidated.

As an important substance released from damaged cells, the S100 protein is a low-molecular-weight acidic protein abundant in nervous system tissues. This protein family was first discovered by Moore [7] and has two calcium-binding sites with EF-hand motif domains. Since S100 proteins are present in systemic organs and can interact with other proteins, they are believed to have a wide variety of functions. S100 proteins alter their morphological form by binding calcium and contacting targeted proteins that influence cellular division [8]. S100 proteins are involved in intra- and extracellular signal transduction [9]. For instance, S100 proteins are released outside the cell and act as ligands for receptors for advanced glycation end product (RAGE), an AGE receptor that subsequently enhances inflammatory reactions as DAMPs.

More than 20 types of S100 proteins have been recognized to date [10] that are widely distributed in various organs. Recent studies have reported their role in human inflammatory diseases. For example, S100A2 is abundantly expressed in epidermal keratinocytes and related to the severity of drug eruption, atopic dermatitis (AD) [11], and the clinical stage of malignant tumors [12]. However, its detailed clinical significance and mechanisms of action remain unclear. To clarify their detailed actions, here, we summarize previous studies of the involvement of S100 proteins in representative inflammatory skin diseases, psoriasis, and AD.

## 2. Pathogenesis of Psoriasis

**Figure 1:**
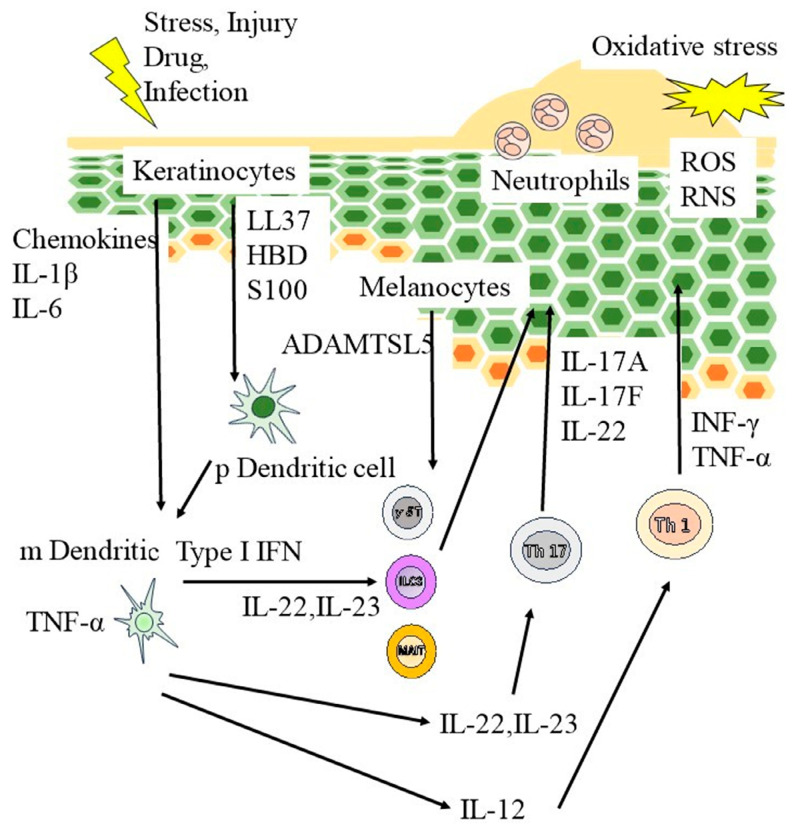
Pathogenesis of psoriasis. External trauma or infection triggers the assembly of host cell–derived nucleotides with keratinocyte-derived antimicrobial peptides to form a complex that triggers the expansion of antigen-specific T lymphocytes in the skin as well as lymph nodes following the activation of antigen-presenting cells. Type I interferons (IFN) are produced by plasmacyte dendritic cells and induce myeloid dendritic cells to secrete interleukin (IL)-23 and tumor necrosis factor (TNF)-α. These cytokines help Th17 cells produce more IL-17 and IL-22, enhancing the inflammatory response in the epidermis and stimulating keratinocyte proliferation. RNS, reactive nitrogen species; ROS, reactive oxygen species.

Psoriasis is a chronic cutaneous inflammatory disease characterized by deregulation of the cutaneous immune system (Figure 1). Scaly erythematous eruptions are a defining feature of the representative inflammatory skin condition known as psoriasis [13]. External trauma or infection triggers the assembly of host cell-derived nucleotides with keratinocyte-derived antimicrobial peptides to form a complex that triggers the expansion of antigen-specific T lymphocytes in the skin and lymph nodes following the activation of antigen-presenting cells [14]. Type I interferons (IFN) are produced by plasmacyte dendritic cells and induce myeloid dendritic cells to secrete interleukin (IL)-23 and tumor necrosis factor (TNF)-α [15]. These cytokines help Th17 cells produce more IL-17 and IL-22, leading to an enhanced inflammatory response in the epidermis and keratinocyte proliferation [13,16]. Specific cytokine inhibitors exhibit potent anti-inflammatory effects against skin inflammation in psoriasis and have demonstrated significance [17]. While the importance of S100 proteins in psoriasis has been gradually elucidated, numerous aspects of the functions of S100 proteins remain unexplained.

## 3. Pathogenesis of AD

**Figure 2:**
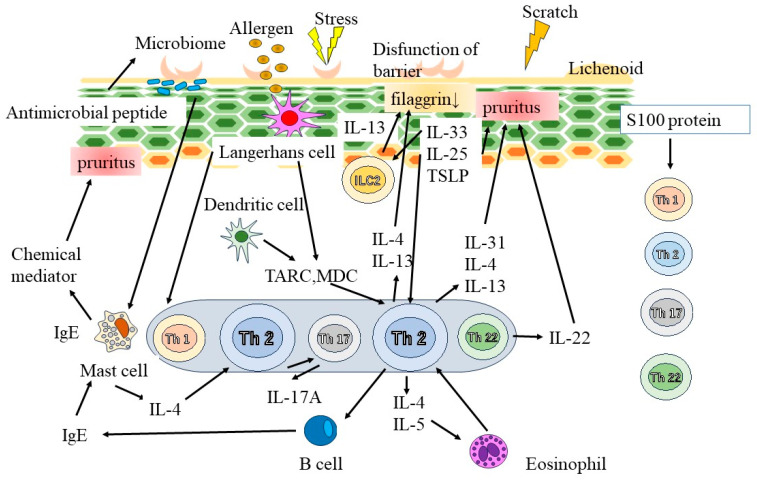
Pathogenesis of AD. The primary components of the skin barrier are intercellular lipids in the stratum corneum, including ceramides. In addition, tight connections illustrate the adhesive structure between the epidermal cells which prevents foreign substrates from penetrating the skin. Chronic skin inflammation in AD is significantly affected by skin barrier dysfunction. Interleukin (IL)-33 and thymic stromal lymphopoietin (TSLP) are produced by epidermal keratinocytes and are responsible for type 2 immune response-mediated inflammation. Thymus- and activation-related chemokine (TARC) as well as macrophage-derived chemokine (MDC) are produced in atopic skin lesions and contribute to the migration of Th2 cells to skin lesions. IgE, immunoglobulin E.

The primary components of the skin barrier are intercellular lipids in the stratum corneum, including ceramides [18,19] (Figure 2). Patients with AD have a reduced stratum corneum ceramide concentration, decreased filaggrin, or loss of function [20]. The epidermis also serves as a crucial barrier to external environmental factors. Tight connections illustrate the adhesive structure between the epidermal cells which prevents foreign substrates from penetrating the skin [21]. Tight connection components are diminished in AD [22], indicating skin barrier disruption in affected patients.

Chronic skin inflammation in AD is significantly affected by skin barrier dysfunction [21]. IL-33 and thymic stromal lymphopoietin (TSLP) are produced by epidermal keratinocytes and are responsible for type 2 immune response-mediated inflammation [23,24]. Thymus- and activation-related chemokine as well as macrophage-derived chemokine are produced in atopic skin lesions and contribute to the migration of Th2 cells to skin lesions [25]. Additionally, IL-22 promotes the development of epidermal thickness in AD [26]. Neutralizing antibody therapy reduces the symptoms of AD and plays a crucial function in the associated itch development [27,28]. Each race exhibits unique immunological characteristics. Asians have a greater proportion of Th17 cells; additionally, an AD animal model [29] suggested that Th17 cells are crucial to the progression of AD.

## 4. S100 Proteins and Psoriasis

In the following sections, we describe the actions of S100 proteins in psoriasis and AD in detail. The currently identified S100 proteins include S100A2, S100A4, S100A7, S100A8, S100A9, S100A11, S100A12, S100A15, and S100B.

## 5. S100A2

Under normal physical conditions, keratinocytes produce dimeric S100A2 [30]. S100A2 is predominantly expressed in human skin; however, its expression is restricted to the basal layer of the epidermis in healthy individuals [11]. In addition, S100A2 is weakly expressed in the cytoplasm, mostly in the nuclei of keratinocytes [31]. In contrast, stress stimuli such as oxidative stress promote cytoplasmic S100A2 translocation from the nucleus, leading to alterations in the distribution pattern of S100A2 in keratinocytes during intracellular Ca^2+^ level elevations [31]. Furthermore, S100A2 is upregulated by IFN [32] and transforming growth factor [33]. As a result, several inflammatory responses appear associated with the pathological inflammatory responses mediated by S100A2. Consistently, S100A2 expression is markedly elevated in the skin of patients with psoriasis [11]. Moreover, S100A2 expression in the skin increases in AD and is associated with disease severity [11]. However, the detailed mechanism of S100A2 action in inflammatory skin diseases remains unclear.

## 6. S100A4

S100A4, a polypeptide that can oligomerize according to the Ca^2+^ concentration, is constructed as an antiparallel homodimer [34]. S100A4 is most frequently found in the cytoplasm of a wide range of cell types. S100A4 is involved in inflammatory reactions [35]. Following certain post-translational changes or stimulation, several investigations revealed its presence in the nucleus [36]. S100A4 can also be released into the extracellular region where it exerts autocrine and paracrine functions [37]. S100A4, when released into the extracellular space attracts immune cells, activates pro-inflammatory pathways and induces cytokine production [38]. Macrophage recruitment to the inflammatory site is consistently impaired in S100A4-deficient mice and these cells exhibit altered chemotaxis and matrix-degrading capacities [39]. Epidermal growth factor receptor, Toll-like receptor 4 (TLR4), annexin-A2, and IL-10 receptor are just a few cell surface receptors with which S100A4 may interact through oligomer formation [40].

S100A4 plays functional roles in psoriasis [41]. It is weakly expressed in the skin, particularly in the upper dermis [41]. S100A4 is predominantly expressed in the upper dermis in psoriasis compared to healthy skin, possibly mediated by p53 [41]. S100A4 inhibitors significantly reduced epidermal hyperplasia and dermal vascularization and impaired keratinocyte proliferation in an animal model of psoriasis [41], suggesting that S100A4 positively regulates skin inflammation in psoriasis.

## 7. S100A7 in Psoriasis

Although the general structure of S100A7 resembles that of other S100 proteins, there are several significant differences, particularly in the *N*-terminal EF-hand motif, which has a twisted loop and lacks a critical calcium-binding residue [42].

Psoriatic skin is prone to higher S100A7 levels in the epidermis [43,44]. The severity of psoriatic inflammation indicated by the Psoriasis Area and Severity Index (PASI) was positively correlated with S100A7 expression in the epidermis, which was downregulated during biological treatment with adalimumab, etanercept, ustekinumab [45,46], or a neutralizing antibody against IL-17A [47]. Consistently, S100A7 is highly expressed in psoriatic plaques with joint involvement [48], indicating its role as a booster of psoriatic inflammation in arthritis.

The binding of S100A7 to RAGE is necessary for inflammatory reactions. S100A7 enhances the chemotaxis of inflammatory cell infiltration into the skin [43]. S100A7 produces IL-1α by keratinocytes in a RAGE–p38 MAPK-dependent manner [47]. S100A7 acts on RAGE and subsequently suppresses involucrin, loricrin, keratin 1, and keratin 10 for keratinocyte differentiation through the MyD88-IκB/nuclear factor-kappa B (NF-κB) signaling pathway [49]. S100A7 activates inflammatory cytokine and chemokine production by neutrophils via ERK and p38 MAPK activation [50].

As the regulatory mechanism of S100A7, IL-17 plays a role in its induction. As the signal pathway for the induction of S100A7 mediated by IL-17, IκBζ is an essential regulatory factor in the pathogenesis of psoriasis through IL-17A and IL-17F-mediated actions [51,52]. Furthermore, IL-17A- and IL-17F-mediated IκBκ activation involves the p38 MAPK and NF-κB cascades [51,52]. IL-17C also has the potential to induce S100A7 expression by keratinocytes [53]. Th17 cells are positively associated with NLRP1-mediated caspase-5 activation during cutaneous inflammation mediated by S100A7 [54]. IL-22 enhances S100A7 expression in keratinocytes and the Janus kinase (JAK) inhibitor tofacitinib has been demonstrated as effective in psoriatic skin inflammation and suppresses IL-22-mediated S100A7 in keratinocytes [55]. In contrast, IL-35 inhibits S100A7 expression in keratinocytes [56]. Peripheral blood mononuclear cells in patients with psoriasis produce S100A7 which increases the production of inflammatory cytokines such as IL-1β, IL-6, IL-8, and TNF-β [57].

Angiogenesis is an essential component of the pathophysiology of inflammatory skin diseases; vascular alterations occur early in the onset of psoriasis [58]. Psoriasis plays a role in angiogenesis hypoxia while reactive oxygen species (ROS) enhance psoriasin expression [59]. The downregulation of S100A7 alters ROS-mediated vascular endothelial growth factor in keratinocytes [59]. S100A7 activates dermal-derived endothelial cell proliferation mediated by the PI3K and NF-kB pathways [59]. Therefore, S100A7 widely affects the pathogenesis of psoriasis and the development of inflammatory reactions within the skin.

S100A7 plays an important role in the pathogenesis of AD. S100A7 is upregulated in AD skin and skin barrier dysfunction upregulates S100A7 in AD skin [60]. S100A7 expression increases in both intrinsic and extrinsic AD skin [61]. Furthermore, the onset of acute AD lesions increases S100A7 production within the skin [62]. Therefore, it is assumed that S100A7 acts in a wide variety of phases and that AD types are involved in its pathogenesis. Regarding the regulatory action of S100A7 in AD, both IL-4 and IL-13 reduced S100A7 [60]. Furthermore, S100A7 in keratinocytes was downregulated by treatment in a JAK2/STAT3-dependent manner, even in the presence of IL-17 [63,64]. STAT3 determines the induction of and influences S100A7 in keratinocytes [63]. JAK inhibitors reduce S100A7 levels in the skin [65]. In contrast, no significant correlation was observed between cutaneous S100A7 expression and the severity of AD skin inflammation or *Staphylococcus aureus* colonization [66]. Therefore, the inflammatory actions of S100A7 may be limited to certain conditions in patients with AD.

## 8. S100A8 and S100A9

The S100A8 and S100A9 subunits, which contain 93 and 114 amino acid residues, respectively, are always bound to one another and modify their self-structures to create polymers in the human body, particularly when Ca^2+^ is present [67,68]. The most common complex was the heterodimer S100A8/A9 [69]. S100A8 is the substance that directly binds to the target molecules whereas S100A9 controls complex activities by guarding S100A8 from degradation, among other actions [70,71].

S100A8 and S100A9 are located in the epidermal suprabasal layers and their expression is negligible in non-lesional psoriatic and healthy skin [72]. Psoriasis severity is associated with S100A8 expression; moreover, S100A9 is highly expressed in psoriatic skin lesions [73]. S100A8 and S100A9 are expressed exclusively in the synovium of patients with psoriatic arthritis (PsA) [74]. In addition, serum S100A8 and S100A9 levels are increased in psoriasis and positively correlated with PASI score [72], indicating that their production is positively correlated with disease development in psoriasis.

Notably, the risk of atherosclerotic events and early cardiovascular disease is associated with skin-derived inflammatory reactions in psoriasis [75]. A lipid-rich necrotic core (LRNC) is a risk factor for coronary plaque development and is linked to future risk of cardiovascular events [76]. Psoriasis severity is associated with LRNC formation and biological treatments can consistently reduce LRNC [77]. S100A8 and S100A9 were reportedly associated with LRNC in patients [75]. A decrease in the LRNCs of S100A8 and S100A9 was observed in patients receiving biological therapy [75]. These findings indicate a potential function of S100A8/A9 in the psoriasis-related formation of coronary plaques, leading to future cardiovascular events.

As a mechanism of S100A8 and S100A9 induction, IL-12/23p40 or anti-IL-23p19 inhibition reduced S100A8 and S100A9 expression in murine skin lesions [78]. Furthermore, IL-17A and IL-17F increase S1008 and S100A9 productions, mainly during the early phases of keratinocyte development [79]. IL-17A consistently enhances S100A9 production by keratinocytes via IκBζ, which was impaired by tacrolimus [80]. These findings indicate that the induction of S100A8 and S100A9 occurs via the IL-23/IL-17 axis.

In addition, S100A8 and S100A9 are induced by TLR4-mediated pathways. Specific peptide sequences within the second calcium-binding EF-hands trigger a TLR4-mediated inflammatory reaction [81]. The S100A8/S100A9 released at the inflammatory lesion was suppressed by calcium-mediated S100A8/S100A9 tetramer formation, leading to the hiding of the TLR4/MD2-binding site in the tetramer shape [81].

The mechanisms of S100A8 and S100A9 in psoriasis were revealed to some degree in previous studies. Recombinant S100A8/A9 proteins induce the expression of several inflammatory cytokine genes and the growth of keratinocytes [82]. S100A9-specific deficient mice show decreased psoriasis-like skin inflammation caused by imiquimod, which is associated with increased psoriasis-associated inflammatory cytokine production in the skin [79].

S100A8 and S100A9 expressions are increased in AD skin [61,62]. They are highly increased in the skin in pediatric-onset AD persisting into adulthood compared with adult-onset AD [83]. S100A8- and S100A9-treated keratinocytes produced inflammatory cytokines and chemokines mediated by p38 MAPK and ERK, respectively, which were impaired by TLR4 inhibitor treatment [84]. S100A8 and S100A9 impair filaggrin and loricrin expression in keratinocytes, indicating their association with skin barrier function in AD [84]. Furthermore, immune cell infiltration depended on S100A8 in the AD skin [85]. A house dust mite stimulates keratinocytes to strongly upregulate S100A8, which is further upregulated in the presence of IL-17A [86]. JAK inhibitors reduce S100A8 in the skin [65]. Emollients also have the potential to reduce S100A8 expression in the skin of AD model mice [87], suggesting that AD treatment may reduce the influence of S100A8 and S100A9 on skin inflammation in AD.

## 9. S100A11

S100A11 exists as a dimer and each monomer exhibits two EF-hands. The Ca^2+^ binding state to the C-terminal EF-hand leads to exposure of the hydrophobic cleft of the protein which becomes a protein–protein interaction site in S100A11 [88].

The involvement of S100A11 in AD pathogenesis is as follows: IL-4 and IL-13 downregulate S100A11 expression in keratinocytes, which also influences the upregulation of filaggrin and human beta-defensin 3 expression, indicating the role of the skin barrier function of S100A11 [89]. Although the direct influence of S100A11 on psoriasis and AD remains unclear, S100A11 plays a role in suppressing cellular growth in human keratinocytes [90] and is possibly involved in epidermal hyperplasia in these inflammatory diseases.

## 10. S100A12

S100A12 is highly expressed in psoriatic skin lesions [91] and substantially associated with PASI and serum S100A12 levels [92]. Etanercept therapy consistently reduced blood levels of S100A12 [92]. S100A12 is associated with PsA. The inflamed synovium expresses S100A12 in the tissue, which is impaired by psoriasis treatment [93]. S100A12 in the serum was positively correlated with PsA disease activity [93]. In contrast, no significant association was observed between LRNC and serum S100A12 in psoriasis [77], possibly indicating a role for S100A12 in the future risk of cardiovascular events.

The epigenetic regulation of DNA or DNA-binding proteins is essential for determining gene expression [94] and is involved in various inflammatory skin diseases [95]. A genome-wide study identified hypermethylation in CpG islands in psoriatic skin and showed a negative association with S100A12 expression, which is highly elevated in psoriasis [96]. Consistently, TNF-α inhibitor treatment accelerates hypomethylation in CpG islands in S100A12 [96], suggesting epigenetic regulation of S100A12 proteins in psoriasis.

S100A12 is increased in AD skin and even in non-lesional skin compared to healthy subjects [97]. However, the detailed mechanisms of action of S100A12 in psoriasis and AD remain unclear.

## 11. S100A15 in Psoriasis

S100A15 was reportedly overexpressed in psoriasis [43,98]. S100A15 expression is increased in the epidermis of the affected patients [98].

Atherosclerosis is an important issue in psoriasis and S100A15 is associated with atherosclerogenesis. The intima–media thickness of the carotid arteries is associated with S100A15 and PASI scores [99]. Furthermore, S100A15 levels are higher in patients with psoriasis and subclinical atherosclerosis [99], indicating that S100A15 is a particularly helpful marker of subclinical atherosclerosis in patients with psoriasis.

S100A15 is differentially regulated by IL-17A, TNF-α, and IL-22 [100]. S100A15 works with S100A7 to stimulate epidermal keratinocytes to produce TNF-α, IL-6, and IL-8 [100]. S100A15 alone has the potential to increase keratinocyte production of IL-6 [100], indicating the importance of S100A15 in the exacerbation of inflammatory reactions in psoriasis.

## 12. S100B

Patients with psoriasis show higher S100B levels; however, the S100B level is not associated with psoriasis severity [101]. Notably, S100B is highly expressed in the skin of patients with non-lesional psoriasis versus psoriatic lesions [101]. However, the detailed mechanism of action of S100B in psoriasis remains unknown.

## 13. Summary of S100 in Psoriasis and AD

### Cutaneous Expression of S100 Proteins in Psoriasis and AD

Here, we summarized the distribution of S100 proteins in psoriasis and AD. S100 proteins are widely distributed in the skin. The basal and spinous layers of the healthy epidermis contain S100A7 and S100A11. The basal layer of keratinocytes contains these proteins in the nucleus and cytoplasm. In addition, these proteins are located in the cytoplasm alone in the spinous layer of keratinocytes [102]. S100A10 is found in the cytoplasm of basal and spinous cells and connected to the plasma membrane [102]. In the healthy epidermis, S100A8 and S100A9 are either missing or expressed at low levels [102]. S100A10 and S100A11 levels in the relevant psoriatic tissues were unaffected whereas S100A7, S100A8, and S100A9 were highly expressed [102]. In psoriasis-affected tissues, S100A8 and S100A9 are highly expressed in the basal and spinous layers [102].

## 14. Regulatory Mechanisms of S100 Proteins in Psoriasis and AD

The regulatory actions of S100 proteins in psoriasis and AD are summarized in Table 1. As a regulatory action of S100 proteins, Th17-associated cytokines activate the expression of S100A proteins whereas Th2-mediated cytokines suppress their expression. IL-4 and IL-13 downregulate S100A7 and S100A15 expression whereas TSLP suppresses S100A8/9 expression. In contrast, IL-17A and IL-22 upregulate the expressions of S100A7, S100A8/9, and S100A15.

## 15. Detailed Action of S100 Proteins in Psoriasis and AD

A comprehensive understanding of S100 proteins in psoriasis and AD is summarized in Table 2. Inflammatory cytokine production has been widely observed in AD and psoriasis as a function of S100 proteins. S100A4, S100A7, S100A8, S100A9, and S100A15 are representative inflammatory cytokines. Keratinocyte growth was observed in S100A8, S100A9, and S100A11. Immune cell infiltration involves the S100A4, S100A8, S100A9, and S100A15 proteins. Angiogenesis has a unique function in S100A7.

## 16. Conclusions

Although the primary source of inflammatory triggers in psoriasis remains unclear, substances released from keratinocytes are considered essential for the onset of psoriasis and AD. Among multiple causal factors, S100 proteins, particularly those in keratinocytes, are considered the main positive drivers of the pathogenesis of psoriasis and AD. This review also demonstrated the complex relationship between S100 proteins and psoriasis-mediated inflammation in other organs and conditions such as PsA and cardiovascular disease. Therefore, larger cohort studies should ascertain whether S100 protein production is associated with psoriasis and AD.

One of the major challenges for physicians is determining the best options for treating psoriasis and AD skin inflammation. The existing information derived from previous studies found that cytokine-targeted therapy displayed excellent therapeutic effects; however, the entire influence of these options for treatment may not be seen in all of these situations. Therefore, an unknown etiology of the development of psoriasis is necessary to unravel the unidentified influences of psoriatic skin inflammation and S100 proteins might be a clue to answer the unknown etiology of the pathogenesis of these inflammatory diseases.

## Figures and Tables

**Table 1:** Regulatory factors of S100 proteins.

Factors	Regulatory Action
IL-4	S100A7↓, S100A15↓
IL-13	S100A7↓, S100A15↓
IL-17	S100A7↑, S100A8/9↑, S100A15↑
IL-22	S100A7↑, S100A15↑
IL-23	S100A8/9↑
TNF-α	S100A15↑
TSLP	S100A8/9↓
IFN	S100A2↑
TGF	S100A2↑

IL, interleukin; TGF, transforming growth factor; TNF, tumor necrosis factor; TSLP, thymic stromal lymphopoietin.

**Table 2:** Regulatory factor of S100 proteins.

S100 Protein	Detailed Action
S100A4	Leukocyte recruitment Inflammatory cytokine production
S100A7	Keratinocyte differentiation Inflammatory cytokine production Angiogenesis
S100A8	Inflammatory cytokine production Keratinocyte growth Impaired filaggrin and loricrin expression
S100A9	Inflammatory cytokine production Keratinocyte growth Impaired filaggrin and loricrin expression
S100A11	Keratinocyte proliferation
S100A15	Inflammatory cytokine production

## Data Availability

Not applicable.
